# ‘Cannibalism’ of exogenous DNA sequences: The ancestral form of adaptive immunity which entails recognition of danger

**DOI:** 10.3389/fimmu.2022.989707

**Published:** 2022-12-23

**Authors:** Christian A. Devaux, Pierre Pontarotti, Sephora Nehari, Didier Raoult

**Affiliations:** ^1^ Aix-Marseille University, Institut de recherche pour le développement (IRD), Assistance Publique Hôpitaux de Marseille (APHM), MEPHI, Institut Hospitalo-universitaire (IHU)-Méditerranée Infection, Marseille, France; ^2^ Department of Biological Sciences, Centre National de la Recherche Scientifique, Centre National de la Recherche Scientifique (CNRS)-SNC5039, Marseille, France

**Keywords:** adaptive immunity, DNA cannibalism, transposons, recombination activating gene (RAG), defective proviruses

## Abstract

Adaptive immunity is a sophisticated form of immune response capable of retaining the molecular memory of a very great diversity of target antigens (epitopes) as non-self. It is capable of reactivating itself upon a second encounter with an immunoglobulin or T-cell receptor antigen-binding site with a known epitope that had previously primed the host immune system. It has long been considered that adaptive immunity is a highly evolved form of non-self recognition that appeared quite late in speciation and complemented a more generalist response called innate immunity. Innate immunity offers a relatively non-specific defense (although mediated by sensors that could specifically recognize virus or bacteria compounds) and which does not retain a memory of the danger. But this notion of recent acquisition of adaptive immunity is challenged by the fact that another form of specific recognition mechanisms already existed in prokaryotes that may be able to specifically auto-protect against external danger. This recognition mechanism can be considered a primitive form of specific (adaptive) non-self recognition. It is based on the fact that many archaea and bacteria use a genome editing system that confers the ability to appropriate viral DNA sequences allowing prokaryotes to prevent host damage through a mechanism very similar to adaptive immunity. This is indistinctly called, ‘endogenization of foreign DNA’ or ‘viral DNA predation’ or, more pictorially ‘DNA cannibalism’. For several years evidence has been accumulating, highlighting the crucial role of endogenization of foreign DNA in the fundamental processes related to adaptive immunity and leading to a change in the dogma that adaptive immunity appeared late in speciation.

## Introduction

Exocannibalism (from Greek *exo-*, ‘from outside’ and cannibalism, ‘to eat humans’), is the consumption of flesh outside one’s close social group. Why introduce the concept of DNA ‘cannibalism’ to describe endogenization of foreign DNA? Over the past centuries, archaeologists and anthropologists have provided evidence of human cannibalism, a primitive behavior consisting of eating one’s own kind. Although cannibalism may have been practiced for different reasons, one of the best-known forms of human cannibalism took place in the context of warfare, either to fully eradicate enemies in a show of anger or to acquire the powers of those who had been defeated (1). Cannibalism is currently a major human taboo, but it is surprisingly common in the animal kingdom and there is a lot of good reasons to eat your own kind (2–4). In this paper the reference to the concept of ‘cannibalism’ is only a metaphor, voluntarily chosen to image a situation of ‘genetic eating of the enemy in a context of warfare’. It illustrates one way among others to evade dangers in the more global context of the fundamental belief that the immune system is designed to discriminate some self from some non-self, especially when the non-self is a pathogenic agents. ‘*Stricto sensu*’ when one evokes a phenomenon of capture by one species (e.g., humans) of DNA sequences relating to a foreign species (e.g., virus), the word ‘cannibalism’ is not appropriate insofar as cannibalism refers to events which occurs between individuals of the same species (consumption of conspecifics as food). It is however worth noting that once endogenized, certain DNA sequences can make it possible to fight against enemies belonging to the species from which they originate. By analogy with primitive human cannibalism, it can be considered that a ‘form of cannibalism’ existed as early as prokaryotes, which have managed to appropriate the ‘strength of their aggressor’ and ‘fight’ against its related organisms through ‘cannibalism of enemy DNA sequences’. Seen under this prism, it is interesting to wonder about the role that DNA ‘cannibalism’ may have played in the conservation of specific defense mechanisms during speciation of both prokaryotes and eukaryotes, and on the mechanisms of DNA sequence transfers (e.g., horizontal transfer, vertical transfer).

Obviously, this concept of DNA ‘cannibalism’ may be surprising for immunologists who advocate: i) that the immune system’s (IS) primary driving force is not to discriminate between self and non-self (complicated structures that often share features with self antigens) but that its function mainly entails recognition of danger; ii) that IS maintains a state of tolerance to pathogen load without compromising host fitness; iii) that the pathological outcome of the microorganism-host interaction is determined by the amount of damage to the host with the possible contributions of both the microorganism and the host; and/or iv) that hereditary information is embedded in diverse physical forms (DNA, epigenetic methylation, RNA, proteins, symbionts) representing a continuum of evolutionary qualities (5–7). The outcome of microorganism-host interaction can be either beneficial or detrimental to the microorganism, to the host, or to both. Evolution of life can be guided by the microorganism-host interactions and is contingent on changing hereditary information relayed through time (inherited information). Ancient microorganism-host interactions in which bacteria were incorporated into a primordial host as organelles are likely to have resulted in the evolution of eukaryotes (8). Commensal bacteria maintaining symbiotic relationship with the host provide essential functions to their host such as nutrition, resistance to pathogen colonization and influence of the immune system, thereby being indispensable for the host’s survival (9, 10). There is also evidence obtained in recombinant-activating gene (RAG) deficient mice suggesting that some microbes may benefit from the specific immune responses that they elicit (11). In the ‘damage-response framework’ model (6), a pathogen is defined as a microorganism that is capable of causing damage to a host. Many microorganisms can be considered dangerous, like viruses or bacteria secreting toxins, yet other similar microorganisms are not, like nonlytic viruses or bacteria secreting beneficial nutrient. If a virus infects a cell, replicates without harming the host cell, and moves on, it would not necessarily damage the host cell and there would be no need to eliminate it and it might even carry genes useful to the host cell (e.g. retroviruses jump in and out constantly, sometimes leaving bits of themselves behind). Archea and bacteria are constantly exposed to the invasion by elements such as phages or plasmids (12). In response they have evolved an arsenal of defense systems such as antiphage systems (frequently physically clustered in genomic ‘defense islands’) or defense systems specifically targeting foreign plasmids, that limit the intrusion of foreign elements (13, 14). The existence of a potential ‘Pan immune system’ where the effective arsenal of defense systems is not encoded by the genome of a single microorganism but rather by its pan-genome, has also been postulated (15). As far back in speciation as in the archaea and bacteria, endogenization of foreign DNA sequences was developed as a mechanism of adaptive immunity allowing the prokaryote to recognize and destroy a pathogenic agent (i.e., a bacteriophage) that would have previously infected it, or one of its ancestors. These mechanisms of DNA ‘cannibalism’ are probably much more numerous and useful than what has previously been described. In particular, it is currently well established that endogenous retroviruses (ERVs) are most likely the proviral remnants of ancestral virus infections. Amazingly, these ERVs form multigene families representing about 8%-10% of the human genome (16, 17). They have certainly contributed to the evolution of most organisms in their complex ecosystems, and for some of them, to survive the pressure of pathogenic agents and their genetic drift (18). This view agrees with the endogenization-amplification theory, which postulates that on various occasions exogenous retroviruses have endogenized in animal genomes, with subsequent amplification (19). It has been estimated that the largest human endogenous retrovirus (ERV) family, HERV-K, consists of 1306 copies (20). Interestingly, Old World monkeys possess a few copies of HERV-K, whereas New World monkeys lack HERV-K, suggesting that HERV-K must have entered the human lineage after the split with the New World monkeys but before the split with the Old World monkeys (21). Since humans possess many more copies than Old World monkeys, this indicates that the HERV-K family must have greatly expanded after the human lineage split from Old World monkeys. Moreover, the study of the evolutionary history of HERV-W in humans (213 copies in the human genome), and ERV-W in primate lineages has revealed that closely related species share the same ERV elements at orthologous loci and that the number of shared elements reflects the degree of relatedness between species (22).

## CRISPR-Cas, the genome editing system that confers adaptive immunity to bacteria

On infection of bacteria, bacteriophages enter either a lytic cycle resulting in lysis of bacterial cells and horizontal transmission or a lysogenic cycle characterized by the integration of the prophage into the host genome and vertical transmission. Bacteria use the genome editing Clustered Regularly Interspaced Short Palindromic Repeats CRISPR-Cas, as an immune defense based on recognition of invading lytic bacteriophages. The CRISPR-Cas systems described in archaea and bacteria have been broadly divided into two classes: Class I (divided into types I, III and IV) and Class 2 (divided into types II, V and VI) (23–25). So, when attacked by a bacteriophage, the target bacterium reacts by ‘viral predation/DNA cannibalism’ as it captures small pieces of the viral DNA and inserts them into its own DNA in a particular pattern leading to generating segments known as CRISPR arrays. The CRISPR arrays allow the ‘educated’ bacterium to ‘retain the memory’ of the aggressor, like a genetic ‘fingerprint’ of the invader. These systems are usually composed of multiple CRISPR-associated genes (cas genes) on the bacterial chromosome and CRISP array consisting of unique DNA spacers. The CRISPR-Cas system mediates its immune defense function through three distinct phases: first, the adaptation phase during which Cas proteins (e.g., Cas 1, Cas 2 sometimes associated with Cas 4) select and process DNA fragments from the invader and insert them into the CRISPR array of the host genome leading to the acquisition of new spacers (it is the central element of the adaptive immune response device); second the expression phase during which the CRISPR locus is transcribed into a long pre-crRNA that is cut into different crRNAs corresponding to different exogenous fragments that combine with Cas proteins constituting a surveillance unit inside the bacterium; third, the interference if the bacterium subsequently encounters the same bacteriophage (or its close relatives) again, the crRNAs-Cas complex will bind to specific complementary regions of the exogenous virus DNA. The bacterium then uses an activate nuclease (e.g. class 1, Cas 3, Cas 9, Cas10) to cut the DNA apart, which disables the virus (26). It is likely the most ancestral form of acquired immunity found in most archaea (about 90%) and in many bacteria (about 40%) (27). However, it was also reported that only 0.83% of *Staphylococcus aureus* strains from different geographical regions have type IIA CRISPR-Cas, suggesting that this specific system may result from spontaneous horizontal gene transfer event (28). It has been proposed that the ‘ancestral CRISPR-Cas system’ appeared in thermophilic archaea, then spread to other lineages of archaea and bacteria through horizontal transfers (29). This system then evolved, both at the level of acquisition of immunity and of the genes coding for the proteins in this defense system, explaining why bacteriophage infection is a major driving force for the maintenance of CRISPR-Cas immune system and why there is a strong selective benefits of phage-encoded anti-CRISPR (Acr) genes for both the phage and the host under context where lysogeny is suppressed. It has been suggested that maladaptive type I CRISPR-Cas immune systems (cannot eliminate the invading bacteriophages due to imperfect matching of spacers to the integrated prophage) occur frequently in nature (30). The Cas proteins constituting the molecular machinery of adaptive immunity have nuclease, helicase, and polymerase-like activities. The diversification of the CRISPR-Cas systems is likely to be partly driven by their competitive coevolution with virus (e.g., phage)-encoded Acr proteins that interact with different CRISPR-Cas components, preventing Cas proteins from binding or cleaving phage DNA (24, 26).

The same mechanism was also reported by one of us (DR) in giant viruses, capable of integrating a sequence from a Zamilon virophage in an operon that was named ‘Mimivirus virophage resistance element’ (MIMIVIRE). Indeed, MIMIVIRE confers lineage A strains of Mimivirus resistance to Zamilon infectivity (31). One of us described this as the ‘Eat me cake’ theory, or ‘genetic cannibalism of the enemy’ theory (32). However, the fact that MIMIVIRE represents an adaptive immune system in giant viruses, remains a subject of debate mainly because the corresponding Zamilon sequence appears devoid of distinct flanking sequences that may serve as protospacer adjacent motifs (33). Anyway, cannibalization of ‘enemy DNA sequences’ likely plays a major role in the protection of populations, including humans, against foreign pathogenic agents.

## From speciation to very elaborate adaptive immunity in humans

Through the work of immunologists, it was established that vertebrates, including humans, are remarkably equipped to respond to attacks by invading pathogens, including bacteria, viruses, fungi, and parasites. Defense mechanisms more or less specific for these pathogens have been classified into two main categories of immune responses: the ‘nonspecific’ innate immunity that takes minutes to hours to operate and does not retain a memory of previous responses, and the adaptive immunity that takes days to weeks before being able to operate and engages the pathogen with specificity and immune memory (**Table 1**). There are four main components of innate immunity, which consist of the activation of pre-existent mechanisms: 1) physical or anatomical barriers (e.g., skin, mucosa, tight junctions) and chemical barriers (e.g., mucus, lysozyme, defensin, reactive oxygen species); 2) phagocytosis (i.e., monocytes, macrophages, neutrophils); 3) blood proteins (e.g. activation of complement, lectins, fibrinogen), and 4) inflammation mediators (i.e., macrophages, mast cells, natural killer cells) and inflammation molecules (e.g., production of interleukins, cytokines and interferon). Activation of the innate immune system is initiated by soluble pattern recognition receptors (PRRs) that can be expressed on innate immune cells, bound to the extracellular matrix, or circulate in the blood as soluble molecules (34). By contrast, specific adaptive immunity depends upon the somatic diversification of antigen-receptor genes to generate vast repertoires of B- and T-cell receptors (35–38). Elegant studies from evolutionary immunologists have revealed that innate immunity can be found within the phylum of multicellular organisms (metazoans) that emerged as long as 600 million years ago followed by a remarkable diversification of metazoan species (including the vertebrate lineage) while adaptive immunity is only evidenced with the gnathostomes (vertebrates with jaws) and evolved to work in concert with the innate immune system (39–42).

**Table 1:** Examples of the outcomes of host defense against microorganisms.

Defense system	Host benefit	Retains the 'memory' of invader
In bacteria		
-Innate immunity		
Phage infection sensor (e.g., CapRelSJ46)	Yes	No
-Adaptive defense		
CRISPR-Cas adaptive immunity	Yes	Yes
In humans		
-Innate immunity		
Defensin	Yes	No
Interferon	Yes	No
-Adaptive defense		
B-cell response	Yes (most frequently)	Yes
Horizontal transfer of transposon	Yes (most frequently)	Yes (most frequently)
Endogenization and provirus 'cannibalism'	Yes (most frequently)	Yes (most frequently)

## The mobile elements of the genomes at the origin of adaptive immunity

It has been proposed that the genes encoding the Cas1 and Cas9 proteins which are experts in cutting and integrating DNA fragments, originate from genes carried by transposons (43, 44). Comparative genomic analysis of Cas1 homologs that are not associated with CRISPR-Cas loci led to the discovery of a novel family of self-synthesizing transposons, the Casposons (e.g., Casposon-encoded Cas 1 or casposase) (24). It is striking to note that Variable/Diversity/Joining (V(D)J) recombination, which makes it possible to create a great diversity of immunoglobulins (>10^14^ different paratopes from immunoglobulins also known as antibodies generated by B-cells) and T-cell receptors (T-cell receptors recognize peptide fragments presented by the major histocompatibility complex class I or class II molecules) in gnathostomes, is based on Rag (recombination activating gene) 1 and Rag2 proteins of the recombinase complex that found their origin in genes carried by a transposon.

Transposable elements (TEs) are major components of all vertebrate genomes that cause genomic instability (45). Transposons are mobile DNA segments (or MGE, for mobile genetic elements) that move from one location to another within the host genome. The retrotransposons also called Class I RNA transposons transpose *via* reverse transcription of an RNA intermediate copied back to the DNA form, while Class II DNA transposons move within the host genome *via* an ‘excision-insertion’ mechanism (46, 47).

Although it was proposed that RAG1 could derive from a herpes virus recombinase or a retroviral nuclease (48, 49), it is more generally accepted that RAG1 derives from a Transib superfamily transposon present in an ancestor of jawed vertebrates that lived about 500 million years ago (50, 51). The ancestral RAG transposon consisting of recombination signal sequences flanking RAG1-like and RAG2-like genes was probably inserted into an exon of a jawed vertebrate ancestor Ig/TCR-like gene. Structural analysis has identified two jawed-vertebrate-specific adaptations that allowed the domestication of ancestral RAG transposase into a RAG recombinase (52). Retention of the RAG1 and RAG2 genes in gnathostomes and duplications of the V, D, and J segments likely built the ancestral recombinatorial immune system of vertebrates. Expression of the recipient gene occurred when the inserted transposon was excised by the Rag proteins and the two exon ends rejoined by double-strand DNA break repair factors. A second transposon insertion into the same exon could have again split this gene into V and D fragments to yield the tripartite V(D)J structure characteristic of the Ig/TCR variable-region genes. In this case it cannot be referred to as DNA ‘cannibalism’ but more simply the domestication of a transposon, except to put forward the unlikely hypothesis that it could be the fingerprint of an ancestral CRISP-Cas cannibalism of RAG to defend a vertebrate against a transposon (53).

## RAG genes and the repertoire of paratopes

In humans, the receptors for non-self epitopes (e.g. immunoglobulin paratopes) are composed of the so-called constant regions and the so-called variable regions that consists of V, D, and J segments. The variable regions are at the origin of the diversity of the paratopes. The V(D)J recombination can be divided into three steps. It is initiated by the Rag1 and Rag2 complex which introduces a double-strand DNA break (dsDNAb) at the border between V and D segments or D and J segments and their respective recombination signal sequences (RSS) that flank each gene segment and that are composed of conserved heptamer and nonamer sequences separated by a poorly conserved spacer sequence of either 12 or 23 bp, creating hairpin-sealed coding ends and blunt signal ends. This DNA lesion is repaired using the non-homologous end-joining (NHEJ) pathway DNA repair machinery. SNM1C/Artemis, which is recruited and phosphorylated by the Ku/DNA-PK complex, opens the DNA hairpins through its endonuclease activity (required for cleavage of hairpin intermediates). The XRCC4/Cernunnos/DNA-LigaseIV complex finally seals coding and signal joins (54–56). This highlight the crucial role of the transfer of the RAG transposons for the generation of the pathway leading to the paratopes repertoire in humans. Domestication of a transposon to give rise to the Rag1-Rag2 recombinase and V(D)J recombination, was a pivotal event in the evolution of adaptive immune system of jawed vertebretes (52). It was recently hypothesized that an ancestral form of the current RAG recombinase function may have been domesticated for the purpose to protect the host against transposons that could jeopardize the integrity of the genome (53).

## Adaptive immunity in multicellular organisms before gnathostomes with jaws: that’s the question

All extant jawed vertebrates can rearrange antibody and T-cell receptor gene segments. The common ‘use it or lose it’ theory (57) assumes that the size of genomes is not infinitely expandable, and species have to make choices. Accordingly, during evolution a ‘useful mechanism’ is generally preserved but can be eliminated from a species if it becomes useless or can be replaced by a more efficient mechanism. So, should we truly consider, as is currently the case, that there was no adaptive immunity in multicellular organisms before gnathostomes? that is all the more surprising since we now know that the first forms of adaptive immunity could be found in unicellular organisms that were present on our planet a billion years ago. Would this adaptive immunity of the first unicellar organisms have lost its usefulness with the establishment of a more effective innate immunity in metazoans or was unfavorable to the survival of multicellular organisms? Vertebrates have always been colonized by bacteria and other microorganisms (microbiota) that perform essential functions for the survival of their host (58). Under these conditions, horizontal transfer of DNA sequences is possible. Because CRISPR comes from bacteria that live on or infect humans, an important fraction of the human population has built up immunity to the Cas9 from bacteria over time (59). Yet for multicellular organisms, cells taken individually are sometimes the target of aggression, such as a viral attack that could lead to an integration of the genome of infectious agents into the host genome. That can be fatal to the host but sometimes gives the host a better adaptive value. Viruses can be exquisite vectors for shuttling foreign DNA into eukaryotic cells and favoring DNA endogenization. This is also true for vertebrates without jaws. Moreover, it has been reported that in the chicken, immunoglobulin light chain (IgL) diversity is generated by recombination between a single functional variable (VL) and joining (JL) gene segments and subsequent somatic diversification of the rearranged VL region (60, 61). The IgL loci of different birds (such as the quail, duck, pigeon, or turkey) consist of a family of VL elements but undergo a single major rearrangement event similar to that observed in chickens. In contrast, several rearrangements have been observed in the Muscovy duck locus the genome of which contains 2 functional VL segments (VL1 and VL5) and 3 VL pseudogenes critical for antibody diversity suggesting that combinatorial IgL diversity has evolved independently in some avian species (62). Interestingly, the RAG1 and RAG2 genes have been found in the chicken but only RAG-2 mRNA expression occurs in B-cells undergoing antibody diversification by gene conversion (63). The study of *Carcharhinus* sharks genome also found the RAG-1 gene in this species with similarities to integration host factor genes of the bacterial site-specific recombination system (64). Thus, the evolution of the ancestral adaptive immune system may have been initiated by a transfer of microbial site-specific recombinases.

Considering these arguments, it is worth noting that the sequence encoding proteins *SpRag1L* and *SpRag2L* closely related to RAG1/RAG2 has been identified in an invertebrate, the purple sea urchin (an echinoderm) (65). This could represent one of the missing links, considering that major DNA elements required to achieve adaptive immunity preexisted in speciation before gnathostomes. Other echinoderms also appear to carry adjacent RAG1 and RAG2-like genes pair in their genome (51).

There is also an adaptive immunity system in agnathans but it is not achieved using RAG system. Indeed, Cyclostomes, which contain two phyla (hagfishes and lamprey) have their own adaptive immune system based on variable lymphocyte receptors (VLRs) the genes of which diversify somatically through gene conversion (66).

## Horizontal transfer of transposon

Several families of repeat ‘active’ mobile genetic elements and ‘fossil’ of previously mobile genetic elements can be found within vertebrate genomes, having been part of our molecular evolution (67–69). In humans they are composed of the long terminal repeat (LTRs) retroelements including the human endogenous retroviruses (HERV) and the retrotransposons, the non-LTR retroelements including the non-autonomous short interspersed elements (SINEs such as Alu and MIR lacking reverse transcriptase) and the long interspersed elements (LINEs such as LINE1), and the DNA transposons. If we can now explain how the system was built that makes it possible to generate adaptive immunity in humans, we do not yet have all the elements necessary to determine when and how the transfer of this genetic information could have occurred. At least seven major classes of DNA transposons are found in the human genome, with some reflecting ancient eukaryotic elements such as the ‘mariner’ sequence (70). Horizontal transmission of a transposon from fish to human has been reported for a member of the Tc1/mariner superfamily of transposons (71). Hundreds of cases of horizontal transfer into new genomic background have been described in multicellular eukaryotes, suggesting that this process has been a major force propelling eukaryotic genome evolution (72). It has been suggested that prokaryotic transposable elements may be delivered to eukaryotic hosts, such as the *IS*5-like insertion sequence transferred into a bdelloid rotifer (73), or the *Merlin* superfamily related to bacterial IS1016 insertion sequences transferred to diverse animal genomes as well as humans (74). This could explain the patchy distribution of some eukaryotic DNA transposons that are phylogenetically related to bacterial insertion sequences. Yet the precise mechanisms by which the transposable element can be transferred from a prokaryote to humans remains largely unknown. It can include potential infectious vectors as suggested by the discovery of a *Bov-B*-derived short interspersed element (a non-LTR retrotransposon) from Echis ocellatus reptiles integrated into the genome of the taterapox virus (TATV), and orthopoxvirus that replicates in a West African rodent (75). LTR retrotransposons can make their own virus-like particles, and sometimes they encode envelope-like proteins (76), that may confer infectious properties facilitating transfer. A recent study has demonstrated the impressive extent of horizontal gene transfer (HGT) between 201 eukaryotic and 108,842 viral taxa with the identification of 1,333 candidates for virus-to-eukaryote transfers, 4,807 candidates for eukaryote-to-virus transfers, and 600 transfers with unknown directionality, altogether affecting 2,841 distinct protein families (77). Thus, the involvement of microorganisms and viruses to explain the ‘cannibalism’ of small DNA sequences is easy to imagine.

**Figure 1:**
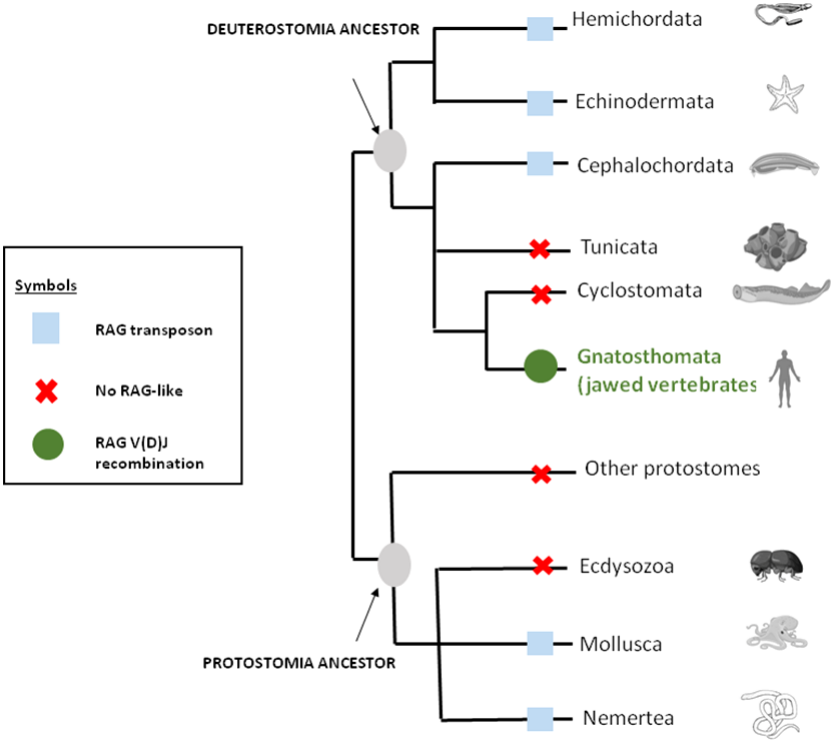
RAG through the eukaryote living world. The distribution of RAG across bilateral metazoans shows that RAG is originally a transposon that was domesticated in jawed vertebrates or gnatosthomes (gnatosthomata).

**Figure 2:**
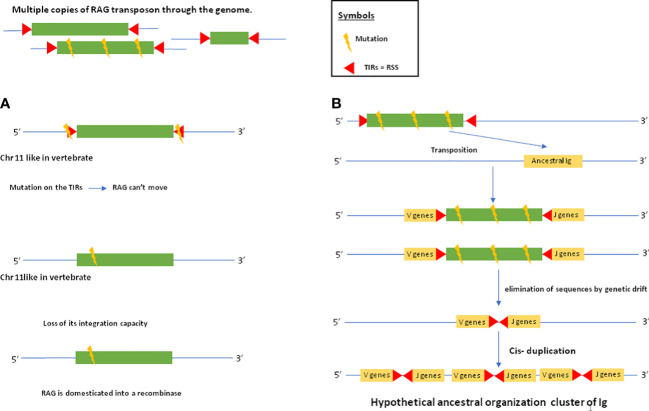
RAG domestication and establishment of the ancestral immunoglobulin cluster. The following evolutionary scenario is assumed: there are several copies of the RAG transposon in the genome. Some of these copies are mutated. In **(A)**, a copy of the RAG transposon is inserted into a chromosome that will later become chromosome 11 in humans where the RAG recombinase is located. This copy of the transposon will be mutated at the level of its TIRs, which will prevent it from jumping elsewhere in the genome. Another mutation within the transposon sequence will cause it to lose its ability to integrate. Thus, the RAG transposon will be domesticated into a recombinase. In **(B)**, a mutated copy of the RAG transposon will insert itself into an ancestral immunoglobulin. This copy is so mutated that the transposon sequence is eliminated by genetic drift, leaving only the TIRs in the ancestral immunoglobulin gene. After cis-duplication, the IG cluster is established. Thus, in jawed vertebrates, domesticated RAG will act in trans by recognizing TIRs that have become RSSs allowing it to rearrange the V,D and J gene segments.

As mentioned above, genes closely related to RAG1 and RAG2 found in echinoderm (51, 65) could correspond to a primitive Rag1/2 but their relationship to transposons is uncertain since this gene pair apparently lacks the terminal invert repeat (TIR) and target site duplication (TSD) signatures of transposons. One of us (PP) reported evidence suggesting that the RAG transposon was active through the deuterostomes evolution and is still active in several lineages (78). (**Figure 1**) This could represent one of the missing links considering that major DNA elements required to achieve adaptive immunity preexisted in speciation before gnathostomes. More recently, RAG-like (RAG-L) transposons were found in protostomes including oysters and mussels, suggesting their ancient bilaterian origin (79) The genetic mechanism of domestication of RAG is shown in **Figure 2**.

## Endogenization and/or provirus DNA ‘cannibalism’

Another form of DNA ‘cannibalism’ is the endogenization of retroviruses. Although infection of humans by retroviruses such has the human T lymphotropic virus (HTLV) or human immunodeficiency virus (HIV) generally has a deleterious effect on the host as a consequence of provirus integration and provirus gene expression, the discovery that some retroviral genes integrated into the DNA of germ-line cells (followed by their vertical transmission from mother to child) can ensure essential biological functions has changed our view about the integration of exogenous viruses and their relationship to adaptive immunity. A common and elegant example of this mechanism can be illustrated by the synthesis of syncytin, an endogenized retroviral envelope protein involved in human placental morphogenesis (80, 81). The comparison of the sequences of syncytin genes in different mammals (primates, ruminants, rodents, etc.) has shown that they come from different retroviral integrations in the genome of the ancestors of each lineage at different times of radiation (diversification) of mammals. The oldest syncytin gene known to date is the syncytin-Car1 gene, found in all carnivore species studied. Its presence would date from at least 80 million years ago (Myr) The integration of the human syncytin 1 gene, conserved in hominoids, would date from 30 Myr while the syncytin 2 gene, present in all primates except prosimians, would be older (45 Myr) The murine syncytin A and B genes would have been integrated more than 25 Myr ago. Thus, retroviral infections have made it possible, on multiple occasions during evolution, to confer a selective advantage on infected individuals of different species, by promoting cell fusion and the formation of a syncytium at the feto-maternal interface and hence embryo development (82, 83).

Since retroviral DNA ‘cannibalism’ has been demonstrated, it can be questioned whether retroviruses that have recently emerged and spread in human populations could be endogenized to provide adaptative immunity to the host against the exogenous retrovirus. Despite the fact that that the HTLV-2 genome has not been found integrated into the germinal cells, the low pathogenic HTLV-2 has been considered to be a good candidate for viral endogenization (84, 85). The question has been addressed regarding the endogenization of HIV (86) and this attractive hypothesis requires further clinicial attention and biological investigation. It was also found that human testicular germ cells can support HIV entry and integration, which could be endogenized in the future (87). Evidence has been reported in animals indicating that this process is currently ongoing with the KoRV retrovirus causing lymphoid neoplasia and immunosuppressive (AIDS-like) disease in koalas (88–90). Following the endogenization process of KoRV-B, the ‘cannibalized KoRV-B DNA’ dam transmission of KoRV-B was demonstrated by the observation that the progeny of koalas are no longer susceptible to the virus (91, 92). Similar observations have been reported for murine leukemia virus (MLV) and mouse mammary tumor virus (MMTV) endogenous in mice (93). Endogenization of MMTV-like elements has been reported in genomes of American pikas (94) or endogenous jaagsiekte sheep retrovirus (enJSRV) in ovine and caprine genomes (95).

## DNA ‘cannibalism’ of provirus

**Figure 3:**
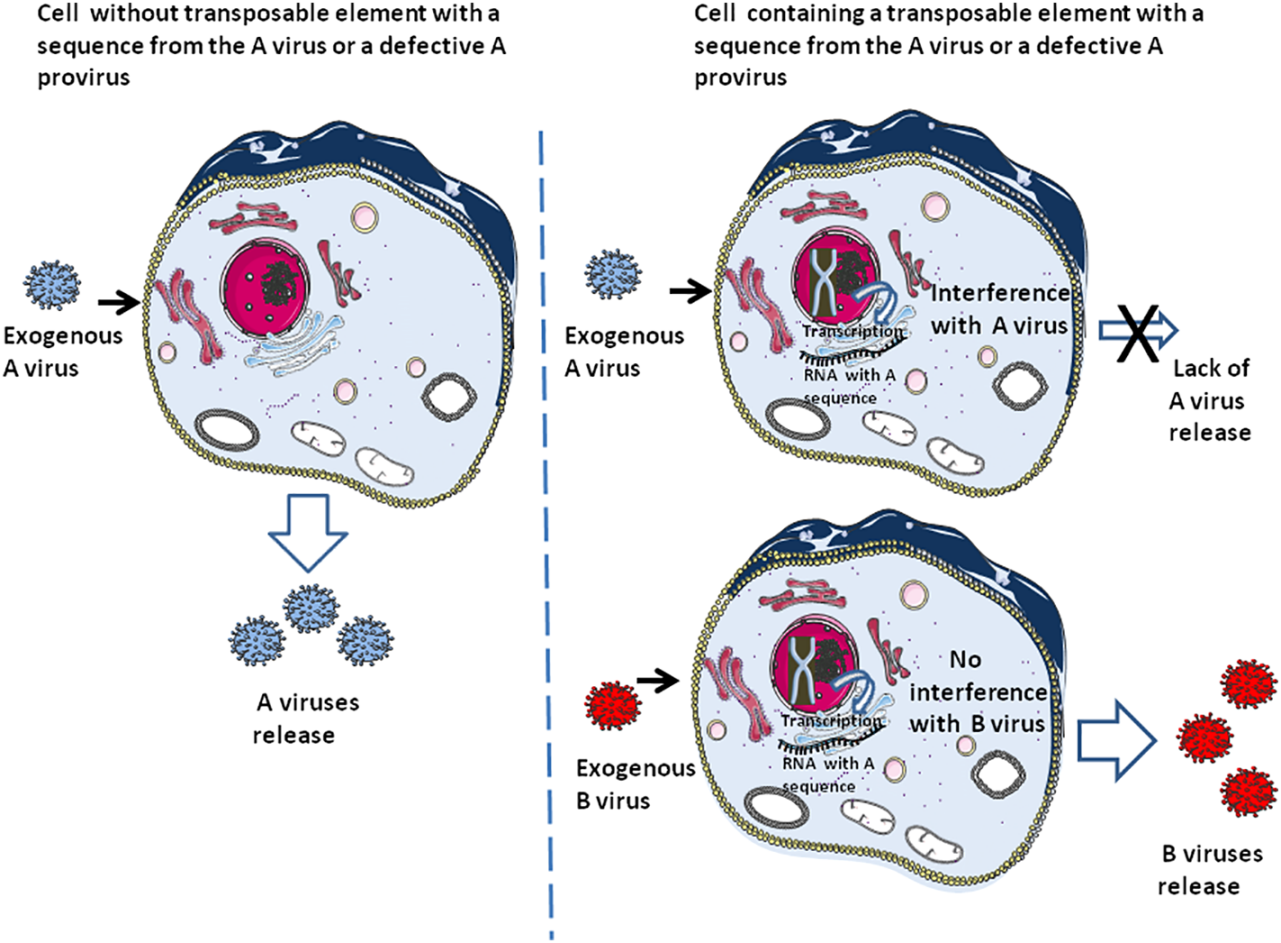
The earliest form of adaptive immunity. This schematic representation illustrates the fact that cells containing transposons carrying a DNA sequence from virus A or an integrated defective A provirus can be protected against an exogenous A virus while remaining susceptible to infection by an exogenous B virus. Due to this selectivity, this form of ‘DNA cannibalism’ can be considered to represent a particular form of adaptive immunity. At the same time, the sensing molecules can recognize the exogenous B virus and initiate an innate immune response (e.g., interferon production).

Defective viral genome (DVG) that produce defective interfering particles containing a fraction of the viral genome are only able to replicate in the presence of a helper virus and can interfere with the replication of homologous infectious viruses containing their full-length genome (96–100). It is thought that DVG may function in the establishment of virus persistence (101, 102). This defective provirus can also be considered as a process related to DNA ‘cannibalism’, conferring either innate immunity (i.e., through induction of interferon), specific adaptive immunity to the host cell against the homologous infectious virus (or the host species when present in the germinal cells), or both (**Figure 3**). This ‘DNA cannibalism’ could be regarded as a provirus DNA ‘cannibalism’ since in this case it is an infectious virus from which part of the proviral genome has been deleted that is endogenized. Regarding the human retrovirus, HTLV-1 is known to generate numerous defective viruses, and within such defective viruses a majority had only one Long terminal repeat (LTR), and the 5’LTR was preferentially deleted in about 40-50% of lymphoma-type adult T leukaemia/lymphoma (103). As reported by one of us (CD), the HTLV-1 HBZ protein, encoded by an anti-sense mRNA, was found expressed in ATL cells carrying defective provirus and is associated with progression to lymphoma (104). Regarding HIV, defective proviruses are produced in large quantities during natural infection. It was found that defective particles do not interfere with virus production from proviral DNA but rather inhibit re-infection, thereby limiting the spread of infection and progression of the disease by reducing the yield of infectious virus (105). Moreover, HIV-1 infectivity and core assembly were reported to be altered due to the interference of HIV-1 Gag formation by HERV-K Gag particles (106) Consequently, post-suicide provirus DNA cannibalism and HERV interference could contribute to generating specific adaptive immunity against infectious viruses.

## DNA ‘cannibalism’ of viral sequences from non-retroviral origin

So far, we have discussed mechanisms involving exogenous retroviruses that have been endogenized or are in the process of endogenizing. But what about viruses that do not belong to the retrovirus family? The main example is the human herpesvirus 6 (HHV-6). HHV-6 infects most people during childhood and can reactivate later in life to cause disease. Both HHV-6A and HHV-6B are, in rare cases, found integrated in leukocyte chromosomes of immunocompetent individuals (107). Sequences homologous to HHV-6 have been found integrated within the genome of about 1% of humans, but it is not clear how this could have occurred. The subtelomeric integration of an ancient HHV6A variant on chromosome 22q (chr22q) in East Asians affects PIWI-interacting RNAs (piRNAs) known to block germline integration of transposons (108).

Adeno-associated virus (AAV), a nonautonomous parvovirus, can establish latency through site-specific genome integration into human chromosome 19 at the *AAVS1/Mbs85* locus involving the AAV2 Rep68 protein (109, 110). Interesting observations have been reported regarding parvoviruses that suggest these viruses have frequently invaded the germ lines of diverse animal species, including mammals, fish, birds and arthropods (111). The identification of orthologous endogenous parvovirus sequences in the genomes of humans and other mammals suggests that parvoviruses have coexisted with mammals for at least 98 Myr. Furthermore, the expression of some of the endogenized parvoviral genes in eukaryotic organisms suggests that these viral genes might have a beneficial function in the host. This indicates that the process of cannibalism is not restricted to retroviruses.

## Discussion

Thus, two apparently very different adaptive immune systems, the ‘old’ CRISPR-Cas system of archaea and the ‘recent’ V(D)J segments rearrangement leading to immunoglobulin and T-cell receptors would have been produced from DNA sequences encoded by transposons (112). However, the precise mechanisms by which transposable elements can be transferred to humans remain largely unknown. It may include potential infectious vectors or be based on the ability of LTR retrotransposons to make their own virus-like particles, which may confer infectious properties facilitating transfer. Thus, the involvement of microorganisms and viruses to explain DNA ‘cannibalism’ of small sequences from exogenous infectious pathogens is currently easier to imagine. The very high percentage of endogenous retroviruses in the human genome suggests that these sequences are likely molecular witnesses of ancestral infections and there is no obvious reason why this process might not reproduce itself in the future with emerging retroviruses or even other human pathogens.

Adaptive immunity has been well characterized in vertebrates with jaws during the past decade. With recent advances in immunology, next generation peptidomics, and genomics it is becoming evident that the T cell receptor (TCR) repertoires of an individual is shaped both by self and non-self antigens (immunopeptides). The impact of the variability in the self-immunopeptidome on thymic selection could explain differences in the TCR repertoire of different individuals, despite an identical HLA type (113, 114). The immunopeptidome includes highly variable sequences such as transposable elements, LINE-1, and endogenous retroviruses (115). The difference in the TCR repertoire driven by the self-immunopeptidome could be important in the initiation of autoimme diseases, cancers and the immune response to pathogens (116, 117). A primitive form of adaptive immunity we called ‘DNA cannibalism’ established in prokaryotes to combat bacteriophages, has also been widely documented. However, there was an apparent gap in between the identification of adaptive immunity in prokaryotes and vertebrates with jaws. Further investigation of these mechanisms in different species progressively allows filling in the gap and arriving at the conclusion that there has been no gap during speciation regarding adaptive immunity. The investigation of the RAG1/RAG2 in humans leads to the discovery of equivalent systems in birds, sharks, and echinoderms and highlights a process of domestication of a transposons during speciation.

There is also evidence that independent events of DNA ‘cannibalism’ in different species may have been functionally convergent in the history of endogenization. If one assumes that adaptive immunity is a general mark of living organisms, it remains to be seen how and when the gene responsible for this function were transferred to the human genome. Arguably the best model for this is the RAG system that has been present in different species during speciation and originate from transposons although it presents homology to microbial integrase. The provirus DNA ‘cannibalism’ found in human appear quite similar to the specific adaptive immunity conferred by ‘DNA cannibalism’ of bacteriophages, as it allows human cells to fight infectious viruses that are homologous to endogenized defective proviruses. However, we know almost nothing about the fact that this process is random or governed by genetics laws.

Until now it has been thought that adaptive immunity should be regarded as a very sophisticated process of recent acquisition complementing innate immunity. In the light of recent data, it has become clear that this is not the case, and that processes leading to various forms of adaptive immunity have existed for a very long time and represent an effective tool both in the specific fight against pathogens and as an active component in the evolution of species.

## Author’s note

We wish to recall that the first references to the word ‘cannibalism’ used as a metaphor intended to image a situation which is not cannibalism *stricto sensu*, are the work of the philosophers Michel Foucault (1926-1984) and Claude Levy Strauss (1908-2009), who held the chair of social anthropology at the College de France and developed the concept of ‘cannibal democracy’.

## Author contributions

CD wrote the first draft of the manuscript. SN designed the figures. All authors contributed to the article and approved the submitted version.
